# Newly diagnosed exudative age-related macular degeneration treated with pegaptanib sodium monotherapy in US community-based practices: medical chart review study

**DOI:** 10.1186/1471-2415-10-2

**Published:** 2010-02-09

**Authors:** Pamela A Weber, Barbara M Wirostko, Xiao Xu, Thomas F Goss, Gergana Zlateva

**Affiliations:** 1 Island Retina, 1500 William Floyd Parkway, Suite 304, Shirley, New York, 11967, USA; 2 Pfizer Inc, 235 East 42nd Street, New York, New York, 10017, USA; 3 Covance Market Access Services, Inc, 9801 Washingtonian Boulevard, 9th Floor Gaithersburg, Maryland, 20878, USA; 4 Current address: Johns Hopkins University, Baltimore, Maryland, USA; 5 Current address: Boston Health Care Associates, Boston, Massachusetts, USA

## Abstract

**Background:**

Studies have shown that early detection and treatment of neovascular age-related macular degeneration (NV-AMD) can delay vision loss and blindness. The objective of this study was to evaluate the efficacy/safety of intravitreal pegaptanib sodium monotherapy in treatment-naïve subjects with newly diagnosed NV-AMD and to gain insight into characteristics of lesions treated in community-based practices.

**Methods:**

From seven private US practices, charts were retrospectively reviewed on 73 subjects with previously untreated subfoveal choroidal NV-AMD treated with their first dose of pegaptanib monotherapy on/after 4/1/2005 through 6/5/2006, receiving ≥4 treatments at 6-week intervals over 21 weeks. Primary endpoint: mean visual acuity (VA) change from baseline to month 6.

**Results:**

75% of lesions were occult, and 82% were subfoveal. From baseline to month 6, mean VA change was -0.68 lines; 58% and 16% gained ≥0 and ≥3 lines of VA, and 70% were responders (<3 lines lost). In 35 subjects with early disease, 80% were responders with a mean gain of 0.46 lines.

**Conclusion:**

Pegaptanib is effective in real-world patients with treatment-naïve NV-AMD in uncontrolled community-based retina practices.

## Background

Age-related macular degeneration (AMD) is a chronic, progressive disease that results in a loss of central vision and significant functional impairment. It is the leading cause of blindness in Western developed countries [1,2]. Neovascular AMD (NV-AMD) represents 10 to 15% of all AMD cases but accounts for 90% of AMD-related severe vision loss [3]. Choroidal neovascularization (CNV) causes disruption of the structure and function of the retinal pigment epithelium and the retinal photoreceptors. Prevalence of late forms of AMD (defined as the presence of NV-AMD or geographic atrophy [4]) increases exponentially with age [5].

Rapid vision loss is a key characteristic of NV-AMD, such that the proportion of untreated patients who develop severe vision loss (> 6 lines) can reach up to 42% in 3 years of follow-up [6]. Additionally, patients with CNV in one eye have an estimated 43% probability of progression to NV-AMD in the fellow eye within 5 years [7]. Patients with visual impairment from AMD lose independence, suffer from fall-related injuries, experience high levels of depression and anxiety, develop annoying visual hallucinations, and require low vision aids [8-12]. Direct medical and non-medical costs and the cost of progression to blindness all contribute to the economic burden of AMD both to the patient and to society. Estimated annual societal costs of all NV-AMD patients in Canada, France, Germany, Spain, and the United Kingdom are substantial, ranging from €671 to €3278 million [13].

Studies show that the early detection and treatment of AMD can delay vision loss and blindness and thus significantly reduce the economic burden of the disease [14,15]. The primary purpose of early treatment ideally would be to defer progression or to promote visual improvement. Prior to the development of anti-vascular endothelial growth factor (VEGF) inhibitors, NV-AMD patients were treated with laser photocoagulation and photodynamic therapy (PDT) with verteporfin (Visudyne^®^). PDT had limited use as it was only approved to treat the predominantly classic type lesion, representing approximately 20% of NV-AMD patients [16] and it merely slowed vision loss [17].

The quest for alternative treatment options in NV-AMD has been precipitated by the increasing prevalence of the disease and by the associated side effects and unsatisfactory outcomes with approved therapies. Anti-VEGF treatments, such as pegaptanib sodium (Macugen^®^), ranibizumab (Lucentis^®^) and bevacizumab (Avastin^®^), are the first pharmacological treatments to address an underlying pathological factor of the CNV of NV-AMD and to address disease progression without healthy ocular tissue destruction. Pegaptanib sodium, a selective anti-VEGF therapy approved for the treatment of all subtypes of NV-AMD, was introduced into the US market in January 2005. Results from phase II/III pivotal clinical trials [18,19] showed that pegaptanib was effective in patients with subfoveal NV-AMD regardless of lesion subtype (i.e. predominantly classic, minimally classic, or occult). Approximately 70% of patients treated with 0.3 mg pegaptanib had stabilised or improved vision (lost < 15 letters [< 3 lines] compared to baseline) at 54 weeks compared to 55% of patients receiving standard-of-care treatment. Additionally, pegaptanib was well tolerated, with the majority of adverse events being ocular in nature and transient.

Currently available efficacy and safety data for pegaptanib are from clinical trials, which may not accurately reflect pegaptanib's real-world use and potential visual outcomes. Further, there is a need to understand which disease characteristics define earlier lesions in order to identify patients who may have a better response to anti-VEFG therapy (i.e. pegaptanib). We performed a retrospective chart review study in newly diagnosed NV-AMD patients initially treated with 0.3 mg pegaptanib in the US to evaluate actual clinical experience with intravitreal pegaptanib monotherapy and to explore the characteristics of lesions in patients in whom a better response to pegaptanib monotherapy was observed.

## Methods

### Study design

This retrospective medical chart review study included 73 newly diagnosed NV-AMD subjects recruited from seven retina specialist offices/clinics in different geographic regions of the US who were treated with pegaptanib monotherapy. Subjects were required to have at least one eye (study eye) that was newly diagnosed with NV-AMD and that was previously untreated for this condition prior to pegaptanib therapy. Subjects had best-corrected Snellen visual acuity (VA) of 20/40 to 20/200 in the study eye when pegaptanib therapy was initiated and were free from any other ocular pathology that would impair VA. Subjects must have received pegaptanib monotherapy for a minimum of four treatments at 6-week intervals over a 21-week period in the study eye, with initial therapy on or after 1 April 2005 through 5 June 2006. All subjects were ≥ 50 years of age and were excluded if they had participated in an investigational drug study within the study period. Site study staff identified potential charts for review according to study inclusion and exclusion criteria and contacted subjects of the potential charts to obtain written informed consent prior to data abstraction. No medical interventions or invasive procedures were required by the study protocol.

This study was conducted according to the tenets of the Declaration of Helsinki. The study protocol and subject informed consent document were approved by the Allendale Investigational Review Board, a central human investigation review board. This research was compliant with Health Insurance Portability and Accountability Act policies and procedures.

### Data abstracted

Abstracted chart data included subjects' demographic characteristics, NV-AMD diagnosis date, VA at the time of NV-AMD diagnosis, comorbid medical conditions, baseline clinical characteristics of the study eye (including VA, angiographic subtype, lesion size, lesion characteristics and lesion location based on fluorescein angiography) prior to pegaptanib monotherapy initiation, date of injection visits, VA assessment at each injection visit and ocular adverse events.

### Endpoints and statistical analysis

The primary endpoint was mean change in best-corrected VA from baseline to month 6. Secondary endpoints included proportions of subjects who gained ≥ 0, 1, 2 and 3 lines of VA and proportion of those who lost < 3 lines, 3 to < 6 lines and 6 or more lines in best-corrected VA from baseline to month 6. Safety analysis was performed based on a pre-specified list of adverse events common with intravitreal injections.

We calculated summary statistics (means and standard deviations [SD] for continuous variables and frequency distributions for categorical variables) to describe sample demographic and clinical characteristics at baseline and clinical characteristics of study eyes at each treatment visit. Mean change in VA from baseline was calculated both in logarithm of the minimum angle of resolution (logMAR) and in line units of VA at each treatment visit and at the month 6 visit minus VA at the baseline visit.

We compared mean change in VA (logMAR; *t*-test or Wilcoxon nonparametric test) and proportions of subjects who gained or lost VA (Fisher's exact test) from baseline to month 6 between those classified as having early lesions and those not having early lesions. Two definitions of early disease (early lesion) from the VISION study [20] were used for the assessment. Definition No.1 defined early disease as a lesion size of < 2 disc areas, baseline VA in the study eye of ≥ 20/80 (≥ 54 Early Treatment of Diabetic Retinopathy Study [ETDRS] letters), and absence of scarring or atrophy within the lesion. Definition No.2 defined early disease as occult with no classic CNV, absence of lipid, and better VA at baseline in the fellow eye (i.e. worse VA at baseline in the study eye). Further, we evaluated subject baseline characteristics and VA change from baseline to month 6 across study sites to assess whether any differences in treatment patterns, subject characteristics or outcomes were observed among sites using *t*-test, Wilcoxon test, or Fisher's exact test, as appropriate.

Statistical significance was evaluated at the 0.05 level with no adjustments for multiple comparisons. All analyses were performed using PC-SAS version 9.1 (SAS Institute, Cary, NC, USA).

## Results

### Baseline demographics and clinical characteristics

Data were collected between August and November 2006 from 73 NV-AMD subjects' medical charts. Subject demographic characteristics and comorbid conditions are summarised in Table 1. The median age of the subjects was 79 years (range, 58-92 years). A majority of the subjects were female (62%) and had at least one cormorbid disease (86%).

**Table 1:** Baseline demographic characteristics and comorbid conditions.

Characteristic	Study sample
Age (years)	
N	73
Mean (SD)	78.3 (7.0)
Median (range)	79 (58 -- 92)
Gender, *n *(%)	
Female	45 (61.6)
Male	27 (37.0)
Missing	1 (1.4)
Ethnicity, *n *(%)	
White, non-Hispanic	26 (35.6)
Missing	47 (64.4)
Reported comorbid disease, *n *(%)	63 (86.3)
Comorbid disease, *n *(%)	
Diabetes	5 (6.8)
Cancer	13 (17.8)
Asthma	4 (5.5)
Chronic obstructive pulmonary disease	2 (2.7)
Arthritis and rheumatism	22 (30.1)
Headache (migraine, cluster)	1 (1.4)
Chronic neck or back pain	1 (1.4)
Heart disease	12 (16.4)
Stroke	2 (2.7)
Sleep disturbance	1 (1.4)
Comorbid disease categories, *n *(%)	
Ocular*	31 (42.5)
Other	55 (75.3)
Comorbid diseases^†^	
N	63
Mean (SD)	3.1 (1.5)
Median (range)	3 (1 -- 7)

* Includes diabetic retinopathy, glaucoma, congenital degeneration of the retina, ocular tumour, cataract, and low vision due to other reasons.

^†^Among subjects with comorbid conditions.

SD = standard deviation.

At baseline, the mean (SD) VA in the study eye was 0.62 (0.24) logMAR, equivalent to approximately 20/80 Snellen, and the mean time from NV-AMD diagnosis to baseline (first treatment) was 2.4 months (Table 2). A majority of subjects had occult lesions (75%); lesion size < 4 disc areas (66%); subfoveal lesion locations (82%). Few subjects had pigment epithelial detachment, retinal angiomatous proliferation, cystoid macular oedema (CME), fibrosis or geographic atrophy. Approximately one-third (34%) had presence of blood reported.

**Table 2:** Baseline clinical characteristics of study eyes.

Characteristic	Study eye
Best-corrected VA (logMAR)	
N	73
Mean (SD)	0.62 (0.24)
Median (range)	0.54 (-0.18 -- 1.00)
Time from NV-AMD diagnosis to baseline, months	
N	73
Mean (SD)	2.4 (5.9)
Median (range)	0 (0 -- 31)
Angiographic subtype, *n *(%)	
Minimally classic	4 (5.5)
Occult	55 (75.3)
Predominantly classic	11 (15.1)
Missing	3 (4.1)
Lesion size (disc area), *n *(%)	
< 2	26 (35.6)
≥ 2 and < 4	22 (30.1)
≥ 4	11 (15.1)
Missing	14 (19.2)
Lesion location, *n *(%)	
Subfoveal	60 (82.2)
Extrafoveal	1 (1.4)
Juxtafoveal	6 (8.2)
Missing	6 (8.2)
Pigment epithelial detachment, *n *(%)	
Absent	46 (63.0)
Present	16 (21.9)
Missing	11 (15.1)
Retinal angiomatous proliferation, *n *(%)	
Absent	58 (79.5)
Present	1 (1.4)
Missing	14 (19.2)
Cystoid macular oedema, *n *(%)	
Absent	52 (71.2)
Present	7 (9.6)
Missing	14 (19.2)
Fibrosis, *n *(%)	
Absent	57 (78.1)
Present	3 (4.1)
Missing	13 (17.8)
Geographic atrophy, *n *(%)	
Absent	54 (74.0)
Present	10 (13.7)
Missing	9 (12.3)
Presence of blood, *n *(%)	
Yes	25 (34.2)
No	43 (58.9)
Missing	5 (6.8)
Estimated percentage of lesion with blood, *n *(%)	
≤ 50%	12 (16.4)
> 50%	3 (4.1)
Missing	58 (79.5)

NV-AMD = neovascular age-related macular degeneration; SD = standard deviation; VA = visual acuity.

### VA change from baseline

#### Overall sample

On average, subjects' VA remained stable through the fourth pegaptanib treatment. There was a slight decline from baseline to the month 6 visit (mean change: 0.07 logMAR [-0.68 lines]; Table 3), with 58% of subjects gaining ≥ 0 lines, 16% gaining ≥ 3 lines, 12% losing > 0 to < 3 lines, and 11% losing ≥ 6 lines. In all, 70% of subjects lost <3 lines of VA.

**Table 3:** Visual acuity (VA; logMAR*) in the study eye by treatment.

Treatment visit	Mean VA		Mean VA change from baseline	
	
	*n*	Mean (SD)	*n*	Mean (SD)
Second treatment	73	0.60 (0.32)	73	-0.02 (0.25) (↑)
Third treatment	73	0.59 (0.28)	73	-0.03 (0.23) (↑)
Fourth treatment	73	0.63 (0.35)	73	0.01 (0.29) (↓)
Month 6 visit	73	0.69 (0.39)	73	0.07 (0.33) (↓)

* ↑ improved; ↓ declined. Negative logMAR changes indicate improvement; positive logMAR changes indicate declination.

LogMAR = logarithm of the minimum angle of resolution; SD = standard deviation.

Evaluation of mean VA change from baseline to month 6 by baseline NV-AMD characteristics showed that only angiographic subtype was significantly associated with VA change: subjects with occult lesions had improvement in mean VA (-0.01 logMAR [0.09 lines]) while those with predominantly classic and minimally classic lesions had a decline in mean VA (0.27 and 0.34 logMAR, [-2.64 and -3.50 lines], respectively; overall p-value = 0.0065 from one-way analysis of variance).

#### By early disease definitions

Eighteen subjects met early disease definition No.1 (lesion size < 2 disk areas, baseline VA of the study eye ≥ 20/80 and absence of scarring or atrophy), and 35 subjects met early disease definition No. 2 (occult with no classic CNV, absence of lipid, and better VA in the fellow eye at baseline). Subjects with missing data who could not be classified with regard to early disease definitions were excluded from the analyses reported below.

**Figure 1:**
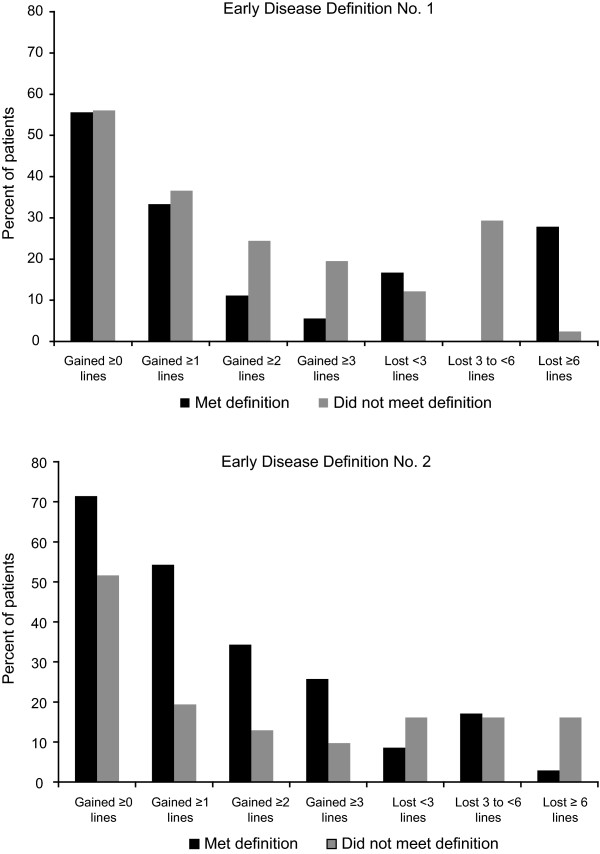
**Distribution of study eye visual acuity change from baseline to month 6 by early lesion definition**.

Subjects meeting early disease definition No.1 showed a decline in mean VA at all treatment visits, and a significant difference in VA change was observed at the third visit relative to those who did not meet the definition (0.08 versus -0.06 logMAR [-0.89 versus 0.61 lines]; p = 0.0283; Table 4). Subjects meeting early disease definition No.2 showed improvement in mean VA from baseline at all treatment visits (p < 0.05 for the third, fourth, and month 6 visits difference between those who met the definition and those who did not meet the definition). Additionally, 26% of subjects meeting early disease definition No.2 had ≥ 3-line gains from baseline to month 6 compared with only 6% among subjects meeting early disease definition No.1 (Figure 1). Using early disease definition No.2, 80% of patients were responders (lost < 3 lines of VA). Mean change in VA from baseline to month 6 was a loss of 0.16 logMAR (-1.61 lines) for definition No.1 and an improvement of -0.05 logMAR (0.46 lines) for definition No.2, with the latter group showing more favourable outcomes compared to the entire sample (0.07 logMAR [-0.68 lines]).

**Table 4:** Mean (SD) change in best-corrected visual acuity (VA; logMAR*) from baseline by early disease definition.

	Met definition		Did not meet definition		
	
Early disease definition	*n*	Mean change (SD)	*n*	Mean change (SD)	p-value
Definition No.1^†^					
Baseline to second treatment visit	18	0.04 (0.33)	41	-0.06 (0.21)	0.1702
Baseline to third treatment visit	18	0.08 (0.29)	41	-0.06 (0.20)	0.0283
Baseline to fourth treatment visit	18	0.12 (0.35)	41	-0.04 (0.26)	0.0597
Baseline to month 6 visit	18	0.16 (0.39)	41	0.03 (0.31)	0.1799
Definition No.2^‡^					
Baseline to second treatment visit	35	-0.06 (0.18)	31	0.03 (0.32)	0.1627
Baseline to third treatment visit	35	-0.09 (0.18)	31	0.03 (0.28)	0.0339
Baseline to fourth treatment visit	35	-0.08 (0.22)	31	0.09 (0.34)	0.0155
Baseline to month 6 visit	35	-0.05 (0.28)	31	0.13 (0.34)	0.0235

* Negative logMAR changes indicate improvement; positive logMAR changes indicate declination.

^†^Early disease definition No.1: lesion size < 2 disk area, baseline VA study eye ≥ 20/80 Snellen, no scar or atrophy.

^‡^Early disease definition No.2: occult and better VA at baseline in fellow eye (worse in study eye).

LogMAR = logarithm of the minimum angle of resolution; SD = standard deviation.

Evaluation of baseline subject clinical characteristics showed that subjects meeting early disease definition No.1 had a mean of 1.1 (median 0.0) months from NV-AMD diagnosis to baseline assessment while those who did not meet the definition for early disease had a mean of 3.1 (median 0.0) months, a difference that did not reach statistical significance in either the mean or the median. Even though more subjects meeting definition No.1 had CME (17% versus 10%, respectively) and pigment epithelial detachment (28% versus 20%, respectively) and fewer were reported to have the presence of blood (22% versus 34%, respectively) compared to those not meeting the definition, none of the differences reached statistical significance (Table 5). Subjects meeting early disease definition No.2 had a mean of 2.5 (median 0.0) months from NV-AMD diagnosis to baseline assessment while those who did not meet this definition had a mean of 0.9 (median 0.0) months (p-values not significant). Even though fewer subjects meeting early disease definition No.2 had CME (3% versus 19%, respectively) or presence of blood (26% versus 48%, respectively) and more subjects had pigment epithelial detachment (31% versus 7%, respectively), only the difference for pigment epithelial detachment reached statistical significance (Table 5).

**Table 5:** Baseline clinical characteristics of study eyes by early disease definition.

	Early disease definition No.1*			Early disease definition No.2^†^		
**Characteristic**	**Met definition**	**Did not meet definition**	**p-value**	**Met definition**	**Did not meet definition**	**p-value**

Best-corrected VA (logMAR), mean (SD)	0.45 (0.10)	0.68 (0.27)	0.0010	0.62 (0.24)	0.64 (0.27)	0.7536
Time from NV-AMD diagnosis to baseline (months), mean (SD)	1.11 (3.12)	3.12 (6.94)	0.2456	2.49 (5.43)	0.90 (2.77)	0.1488
Angiographic subtype, *n *(%)			0.6817			< 0.0001
Minimally classic	1 (5.6)	3 (7.3)		0 (0.0)	4 (12.9)	
Occult	13 (72.2)	33 (80.5)		35 (100.0)	16 (51.6)	
Predominantly classic	4 (22.2)	5 (12.2)		0 (0.0)	11 (35.5)	
Missing	0 (0.0)	0 (0.0)		0 (0.0)	0 (0.0)	
Lesion location, *n *(%)			0.1345			1.0000
Subfoveal	14 (77.8)	38 (92.7)		30 (85.7)	28 (90.3)	
Extrafoveal	1 (5.6)	0 (0.0)		0 (0.0)	0 (0.0)	
Juxtafoveal	2 (11.1)	3 (7.3)		3 (8.6)	2 (6.5)	
Missing	1 (5.6)	0 (0.0)		2 (5.7)	1 (3.2)	
Pigment epithelial detachment, *n *(%)			0.5091			0.0202
Present	5 (27.8)	8 (19.5)		11 (31.4)	2 (6.5)	
Absent	13 (72.2)	33 (80.5)		19 (54.3)	26 (83.9)	
Missing	0 (0.0)	0 (0.0)		5 (14.3)	3 (9.7)	
Cystoid macular oedema, *n *(%)			0.6639			0.0582
Present	3 (16.7)	4 (9.8)		1 (2.9)	6 (19.4)	
Absent	15 (83.3)	37 (90.2)		26 (74.3)	22 (71.0)	
Missing	0 (0.0)	0 (0.0)		8 (22.9)	3 (9.7)	
Fibrosis, *n *(%)			1.0000			0.1585
Present	0 (0.0)	2 (4.9)		1 (2.9)	2 (6.5)	
Absent	18 (100.0)	39 (95.1)		26 (74.3)	27 (87.1)	
Missing	0 (0.0)	0 (0.0)		8 (22.9)	2 (6.5)	
Geographic atrophy, *n *(%)			0.1637			0.2125
Present	0 (0.0)	6 (14.6)		4 (11.4)	5 (16.1)	
Absent	18 (100.0)	35 (85.4)		25 (71.4)	25 (80.6)	
Missing	0 (0.0)	0 (0.0)		6 (17.1)	1 (3.2)	
Presence of blood, *n *(%)			0.5404			0.0806
Yes	4 (22.2)	14 (34.1)		9 (25.7)	15 (48.4)	
No	14 (77.8)	27 (65.9)		24 (68.6)	16 (51.6)	
Missing	0 (0.0)	0 (0.0)		2 (5.7)	0 (0.0)	

* Early disease definition No.1: lesion size < 2 disk area, baseline VA study eye ≥ 20/80 Snellen, no scar or atrophy within the lesion.

^†^Early disease definition No.2: occult and better VA at baseline in fellow eye (worse in study eye).

LogMAR = logarithm of the minimum angle of resolution; NV-AMD = neovascular age-related macular degeneration; SD = standard deviation; VA = visual acuity.

#### By study sites

**Figure 2:**
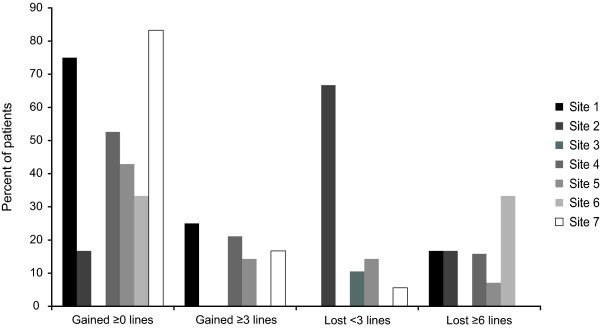
**Distribution of study eye visual acuity change from baseline to month 6 by site**.

When mean VA change from baseline to month 6 was evaluated by study sites, one site (No.7) showed a mean VA gain of -0.04 logMAR (0.50 lines) compared to a mean loss of VA ranging from 0.02-0.30 logMAR (-0.17 to -3.00 lines) for all other sites. Figure 2 shows proportions of subjects with VA change by site. Demographic and baseline clinical characteristics of the subjects were compared between site No.7 and all other sites to explore any subject or practice characteristics that might lead to substantially more subjects treated at site No.7 maintaining or improving their VA from baseline to month 6 compared to other sites (83% versus 49%, respectively; p = 0.0134; Table 6). There was no statistically significant difference observed in subjects' baseline demographic or clinical characteristics between the sites. However, the mean time from NV-AMD diagnosis to initiation of treatment was much shorter for site No.7 subjects compared to that of the other sites overall (0.33 versus 3.13 months, respectively; p = 0.0808), and substantially more subjects at site No.7 did not have geographic atrophy (94% versus 67%, respectively; p = 0.0545).

**Table 6:** Comparison of baseline characteristics and clinical efficacy between site No.7 versus other sites.

Characteristic	Site No.7	Other sites	p-value
Baseline best-corrected VA (logMAR)			
Mean (SD)	0.61 (0.26)	0.62 (0.24)	0.8429
Time from NV-AMD diagnosis to baseline (months)			
N	18	55	
Mean (SD)	0.33 (0.77)	3.13 (6.65)	0.0808
Median (range)	0.0 (0--3)	0.0 (0--31)	
Angiographic subtype, *n *(%)			0.1937
Minimally classic	0 (0.0)	4 (7.3)	
Occult	12 (66.7)	43 (78.2)	
Predominantly classic	5 (27.8)	6 (10.9)	
Missing	1 (5.6)	2 (3.6)	
Lesion size (disc area), *n *(%)			0.1376
<2	9 (50.0)	17 (30.9)	
≥ 2 and < 4	7 (38.9)	15 (27.3)	
≥ 4	1 (5.6)	10 (18.2)	
Missing	1 (5.6)	13 (23.6)	
Geographic atrophy, *n *(%)			0.0545
Present	0 (0.0)	10 (18.2)	
Absent	17 (94.4)	37 (67.3)	
Missing	1 (5.6)	8 (14.5)	
Presence of blood, *n *(%)			0.4277
Present	4 (22.2)	21 (38.2)	
Absent	13 (72.2)	30 (54.5)	
Missing	1 (5.6)	4 (7.3)	
Met early disease definition No.1*	5 (29.4)	13 (31.0)	1.0000
Met early disease definition No.2^†^	5 (29.4)	30 (61.2)	0.0281
VA, baseline to month 6 (logMAR) Mean (SD)	-0.04 (0.22)	0.11 (0.36)	0.0970
VA change, baseline to month 6			
Gained ≥ 0 lines	15 (83.3)	27 (49.1)	0.0134
Gained ≥ 3 lines	3 (16.7)	9 (16.4)	1.0000
Lost < 3 lines	1 (5.6)	8 (14.5)	0.4365
Lost ≥ 6 lines	0 (0.0)	8 (14.5)	0.1871

* Early disease definition No.1: lesion size <2 disk area, baseline VA study eye ≥ 20/80 Snellen, no scar or atrophy.

^†^Early disease definition No.2: occult and better VA at baseline in fellow eye (worse in study eye).

LogMAR = logarithm of the minimum angle of resolution; NV-AMD = neovascular age-related macular degeneration; SD = standard deviation; VA = visual acuity.

### Ocular safety

Pegaptanib appears to be safe for use (Table 7). There was one report of endophthalmitis and two reports of geographic atrophy (occurrence rates of 0.003 and 0.006, respectively, in 326 injections), but the history of these subjects is not known.

**Table 7:** Ocular adverse events over a 6-month period from pegaptanib treatment initiation.

Adverse event	Number of subjects with occurrence	Total number of occurrences	Rate of occurrence by number of injections*
Endophthalmitis	1^†^	1^†^	0.003
Retinal detachment	0	0	0
Increased intraocular pressure	0	0	0
Retinal tear	0	0	0
Traumatic cataract	0	0	0
Vitreous haemorrhage	0	0	0
Geographic atrophy	2^†^	2^†^	0.006
Other	0	0	0

* Based on 326 total injections received by study subjects.

^† ^Unclear history.

## Discussion

The efficacy-related findings of this real-world observational study are similar to those previously reported in pegaptanib clinical trials. In the current study, the mean change in VA from baseline to month 6 was a decline of -0.68 lines (0.07 logMAR), and 70% of subjects lost fewer than three lines. The VISION trials [18] showed a decline of approximately 8 letters, and 70% of subjects lost fewer than three lines of VA after treatment over a period of 54 weeks.

There is no universally accepted definition of early CNV secondary to AMD. The early lesion definitions used in this analysis were based on those used in the exploratory analysis of the VISION study results [20], matching as closely as possible those definitions using the data we had available. Subjects in our study who met early definition No.2 had significantly greater improvement in VA from baseline to month 6 compared to the decline in VA in those who did not meet the definition (-0.05 versus 0.13 logMAR [0.46 versus -1.29 lines], respectively; p = 0.0235). This result was likely driven by the high proportion of patients with occult lesions in the study while few subjects with predominantly classic or minimally classic lesions were included. This suggests that clinicians when selecting out earlier lesions are defining these lesions with occult characteristics. Our current study found that, on average, subjects with occult lesions treated with pegaptanib had an improvement in VA from baseline to month 6 while subjects with predominantly classic or minimally classic lesions had a decline in VA (-0.01 versus 0.27 versus 0.34 logMAR [0.09 versus -2.64 versus -3.50 lines], respectively; p = 0.0065).

In this study, we observed a significant difference in clinical efficacy in subjects across study sites. In particular, 83% of subjects treated at site No.7 showed either an improvement or maintenance of VA from baseline to month 6, with a mean improvement of -0.04 logMAR (0.50 lines), while subjects from all other sites had a mean decline of 0.11 logMAR (-1.07 lines), with only 49% of subjects showing either an improvement or maintenance of VA (p = 0.0134). The earlier treatment of subjects at site No.7 might have accounted for the better results - the mean time from NV-AMD diagnosis to treatment was 0.3 months for site No.7 compared to 3.1 months for all other sites (p = 0.0808). Whether or not anti-VEGF efficacy can be related to the duration of existing CNV disease has not been clearly defined. In a recent study by Boyer et al. [21], efficacy of ranibizumab did not appear to differ across the different quartiles of duration of NV-AMD, which is the opposite of what we found in this study with pegaptanib administration.

Although large randomised, prospective studies have confirmed benefit with treatment, the question remains as to which type of CNV lesions responds best to treatment. Different characteristics were identified in previous studies with no consensus. For example, the TAP [Treatment of Age-related Macular Degeneration with Photodynamic Therapy] and VIP [Verteporfin in Photodynamic Therapy] Study Groups [22] concluded that lesion size was a strong predictor of VA outcome based upon observed PDT treatment and outcome while the VISION trials [20] found that anti-VEGF treatment of early lesions (defined on the basis of the combined characteristics of size, lipid, scarring and time of presentation) had a positive impact on VA outcome. A different conclusion was drawn from the MARINA study [21] subanalysis of subjects with minimally classic and occult lesions treated with ranibizumab in which neither lesion size nor duration of NV-AMD was found to have a direct relationship with VA outcomes. Our study also attempted to evaluate which of the subjects' baseline angiographic characteristics had an impact on VA outcome and found that those who met the early disease definition using the criteria of occult with no classic CNV, absence of lipid, and better VA at baseline in the fellow eye had better VA outcomes. There is no agreed upon and accepted early lesion definition at the present time.

This study intended to evaluate newly diagnosed NV-AMD patients treated with pegaptanib monotherapy. Even though we did not provide participating clinicians with a definition of 'newly diagnosed,' we did not find differences in angiographic characteristics across patients' time since diagnosis. However, we did observe a relationship between time since diagnosis and primary study outcomes of VA change from baseline to month 6 with shorter time being associated with better outcomes. The finding is consistent with the oncology literature that shows that younger tumours that are earlier in their angiogenic process of new blood vessel development appear to be more susceptible to certain types of anti-VEGF therapy [23,24].

There are several limitations to this study. The study included only those practices and patients who were willing to participate. Two sites had a total of four patients who met study inclusion criteria and who were willing to participate, with one patient having maintained VA and the other three having lost more than three lines from baseline to month 6. We cannot be certain that these four individuals are representative of the universe of NV-AMD patients treated in these two practices. Conversely, one does not know how representative patients from site No.7 are to the NV-AMD population either. The sample size of the study limited our ability to perform multivariate analyses that might better support interpreting study findings. In addition, although the proportion of patients losing VA from baseline to month 6 appears to correlate with the presence of classic lesions, this is most likely biased by the fact that our sample included few subjects with classic lesions. Further, the study did not collect optical coherence tomography data for each visit, which might have provided a more accurate summary of NV-AMD characteristics, enabling us to evaluate the relationship between VA change and disease characteristics. Finally, the data were collected for 6 months only; it is not known if the results would have been different over a longer treatment period.

## Conclusion

The efficacy of pegaptanib in community-based practices appears to confirm findings from the VISION trials and a published analysis of patients with earlier disease. There appears to be a trend for patients with earlier lesions to respond more favourably to pegaptanib. Due to our small sample size, there was significant variability of outcomes by site and by patient. Still, shorter time from NV-AMD diagnosis to initiation of pegaptanib treatment appears to be associated with better treatment outcomes and enhanced clinical benefits. It is a common theme across the medical and scientific literature that earlier intervention, prior to permanent damage, is more likely to achieve a beneficial outcome. Other large NV-AMD clinical studies [21,22] have attempted to define and interpret outcome based on lesion characteristics. In this study, though, other disease characteristics did not seem useful in identifying *a priori *responders to treatment. Further research is warranted to fully understand and determine NV-AMD disease characteristics that help predict outcomes. Their results will enable NV-AMD therapy to be targeted to provide the greatest benefit to both patients and society.

## Competing interests

Pamela A. Weber was compensated for her work as an investigator in the study by Pfizer Inc and (OSI) Eyetech, Inc. Barbara M. Wirostko and Gergana Zlateva are employees of Pfizer Inc and own Pfizer Inc stock and stock options. Xiao Xu and Thomas F. Goss were employees (at the time the study was conducted) of Covance Market Access Services, Inc., the company that held the contract for conducting the study. The research was funded by Pfizer Inc and (OSI) Eyetech, Inc.

## Authors' contributions

GZ, BW, XX and TG contributed in the study design, data analysis, data interpretation and writing of the manuscript. PW was a primary study investigator, collected data, contributed to the data interpretation and writing of the manuscript. XX and TG carried out the statistical analysis.

## Pre-publication history

The pre-publication history for this paper can be accessed here:

http://www.biomedcentral.com/1471-2415/10/2/prepub

## References

[B1] Ambati J Ambati BK Yoo SH Ianchulev S Adamis AP Age-related macular degeneration: etiology, pathogenesis, and therapeutic strategies Surv Ophthalmol 2003 48 257 293 10.1016/S0039-6257(03)00030-4 12745003

[B2] Resnikoff S Pascolini D Etya'ale D Kocur I Pararajasegaram R Pokharel GP Mariotti SP Global data on visual impairment in the year 2002 Bull World Health Organ 2004 82 844 851 15640920 PMC2623053

[B3] AMD Alliance International Facts about AMD http://www.amdalliance.org/information/basicfacts/typesofamd.php

[B4] Gottlieb MD Justin L Age-related macular degeneration JAMA 2002 288 2233 2236 10.1001/jama.288.18.2233 12425683

[B5] Friedman DS O'Colmain BJ Munoz B Tomany SC McCarty C de Jong PT Nemesure B Mitchell P Kempen J Prevalence of age-related macular degeneration in the United States Arch Ophthalmol 2004 122 564 572 10.1001/archopht.122.7.1019 15078675

[B6] Wong T Chakravarthy U Klein R Mitchell P Zlateva G Buggage R Fahrbach K Probst C Sledge I The natural history and prognosis of neovascular age-related macular degeneration: a systematic review of the literature and meta-analysis Ophthalmology 2008 115 116 126 10.1016/j.ophtha.2007.03.008 17675159

[B7] Age-related Eye Disease Study Research Group A randomized, placebo-controlled, clinical trial of high-dose supplementation with vitamins C and E, beta carotene, and zinc for age-related macular degeneration and vision loss: AREDS Report 8 Arch Ophthalmol 2001 119 1417 1436 11594942 10.1001/archopht.119.10.1417 PMC1462955

[B8] Dargent-Molina P Favier F Grandjean H Baudoin C Schott AM Hausherr E Meunier PJ Bréart G Fall-related factors and risk of hip fracture: the EPIDOS prospective study Lancet 1996 348 145 149 10.1016/S0140-6736(96)01440-7 8684153

[B9] Ivers RQ Cumming RG Mitchell P Simpson JM Peduto AJ Visual risk factors for hip fracture in older people J Am Geriatr Soc 2003 51 356 363 10.1046/j.1532-5415.2003.51109.x 12588579

[B10] Lee DJ Gómez-Marín O Lam BL Zheng DD Visual impairment and unintentional injury mortality: the National Health Interview Survey 1986-1994 Am J Ophthalmol 2003 136 1152 1154 10.1016/S0002-9394(03)00573-7 14644228

[B11] Lotery A Xiao X Zlatava G Loftus J Burden of illness, visual impairment and health resource utilisation of patients with neovascular age-related macular degeneration: results from the UK cohort of a five-country cross-sectional study Br J Ophthalmol 2007 91 1303 1307 10.1136/bjo.2007.116939 17504847 PMC2000983

[B12] Soubrane G Cruess A Lotery A Pauleikhoff D Monès J Xu X Zlateva G Buggage R Conlon J Goss TF Burden and health care resource utilization in neovascular age-related macular degeneration: findings of a multicountry study Arch Ophthalmol 2007 125 1249 1254 10.1001/archopht.125.9.1249 17846366

[B13] Cruess AF Zlateva G Xu X Soubrane G Pauleikhoff D Lotery A Mones J Buggage R Schaefer C Knight T Goss TF Economic burden of bilateral neovascular age-related macular degeneration: multi-country observational study Pharmacoeconomics 2008 26 57 73 10.2165/00019053-200826010-00006 18088159

[B14] Bonsastre J Le Pen C Soubrane G Quentel G The burden of age-related macular degeneration: results of a cohort study in two French referral centres Pharmacoeconomics 2003 21 181 190 10.2165/00019053-200321030-00003 12558468

[B15] Hopley C Salkeld G Wang JJ Mitchel P Cost-utility of screening and treatment for early AMD with zinc and antioxidants Br J Ophthalmol 2004 88 450 454 10.1136/bjo.2003.035279 15031152 PMC1772079

[B16] Olsen TW Feng X Kasper TJ Rath PP Steuer ER Fluorescein angiographic lesion type frequency in neovascular age-related macular degeneration Ophthalmology 2004 111 250 255 10.1016/j.ophtha.2003.05.030 15019371

[B17] Treatment of Age-related Macular Degeneration With Photodynamic Therapy Study Group Photodynamic therapy of subfoveal choroidal neovascularization in age-related macular degeneration with verteporfin: one-year results of 2 randomized clinical trials--TAP report. Treatment of age-related macular degeneration with photodynamic therapy (TAP) Study Group Arch Ophthalmol 1999 117 1329 1345 10532441

[B18] Gragoudas ES Adamis AP Cunningham ET Jr Feinsod M Guyer DR Pegaptanib for neovascular age-related macular degeneration N Engl J Med 2004 351 2805 2816 10.1056/NEJMoa042760 15625332

[B19] VEGF Inhibition Study in Ocular Neovascularization (V.I.S.I.O.N.) Clinical Trial Group Chakravarthy U Adamis AP Cunningham ET Jr Goldbaum M Guyer DR Katz B Patel M Year 2 efficacy results of 2 randomized controlled clinical trials of pegaptanib for neovascular age-related macular degeneration Ophthalmology 2006 113 1508 1521 16828500 10.1016/j.ophtha.2006.02.064

[B20] Gonzales CR Enhanced efficacy associated with early treatment of neovascular age-related macular degeneration with pegaptanib sodium: an exploratory analysis Retina 2000 25 815 827 10.1097/00006982-200510000-00001 16205558

[B21] Boyer DS Antoszyk AN Awh CC Bhisitkul RB Shapiro H Acharya NR MARINA Study Group Subgroup analysis of the MARINA study of ranibizumab in neovascular age-related macular degeneration Ophthalmology 2007 114 246 252 10.1016/j.ophtha.2006.10.045 17270674

[B22] Treatment of Age-related Macular Degeneration with Photodynamic Therapy (TAP) and Verteporfin in Photodynamic Therapy (VIP) Study Groups Effect of lesion size, visual acuity, and lesion composition on visual acuity change with and without verteporfin therapy for choroidal neovascularization secondary to age-related macular degeneration--TAP and VIP Report 1 Am J Ophthalmol 2003 136 407 418 10.1016/S0002-9394(03)00223-X 12967792

[B23] Benjamin LE Hemo I Keshet E A plasticity window for blood vessel remodeling is defined by pericyte coverage of the preformed endothelial network and is regulated by PDGF-B and VEGF Development 1998 125 1591 1598 9521897 10.1242/dev.125.9.1591

[B24] Marx J Angiogenesis: A boost for tumor starvation Science 2003 301 452 454 10.1126/science.301.5632.452 12881543

